# The Dental Profession of England

**Published:** 1885-09

**Authors:** Felix Weiss

**Affiliations:** London


### THE DENTAL PROFESSION OF ENGLAND.

BY FELIX WEISS, L. D. S., LONDON.

In the issue of the INDEPENDENT PRACTITIONER for July, there is published a communication from Mr. W. H. Waite, of Liverpool, entitled "Some Recent Events Connected with the Dental Profession in England." No one on this side of the Atlantic can have any objection to Mr. Waite's communication, *as far as it goes*, but, unfortunately, the whole truth has not been stated by him. Every dentist here is aware that in July, 1878, an Act was passed through Parliament, and after the 1st of August, 1879, no one could practice unless he possessed a license from one of the medical colleges, or was already on the register. The College of Surgeons of England had its days of grace, and then its doors were closed upon all those who were not prepared to carry out the curriculum. But the other licensing bodies of Great Britain, being equally entitled to confer diplomas, continue to receive candidates to this day, without curriculum, and what is more, when they have passed their light examination they affix to their names the coveted L. D. S., but, in most instances, without the name of the source from whence they derive their qualification.

This requires no confirmation of mine, for everyone knows that the degree given by the Irish college, for instance, subjects its possessor to so little study that its value is simply nominal, and, in proof of this, those members who possess it leave out the last letter, "I," only using the L. D. S. From the earliest period of the existence of the English College, it has been called *The* Royal College of Surgeons. So these letters, without anything added to them, betoken the English title. If the Irish College is proud of the distinction it confers, how is it that the Irish licentiates invariably leave out the "Ireland," or the letter "I"? It is unfair to those who hold the diploma of the English College, and who gained it by passing the curriculum, to class them with men who had simply to go to Dublin, pay their fees, and receive their certificate, although these gentlemen were in practice before the passing of the Act. So long as the Irish College keeps its doors open, so long will the L. D. S. of Ireland continue to possess little value. Curriculum men have had to work assiduously for this distinction; they have not only worked hard, but they have been put to much expense, and it is an act of gross injustice to try to place others on an equal footing who have neither had to study nor to pass any examination worth speaking about. In other respects, Mr. Waite's communication fairly represents "the dental profession in England."

LONDON, July 16, 1885.

